# Smartphone addiction and academic procrastination among college students: a serial mediation model of self-control and academic self-efficacy

**DOI:** 10.3389/fpsyt.2025.1572963

**Published:** 2025-05-29

**Authors:** Xiuli Zhao, Huahua Wang, Zaoming Ma, Libing Zhang, Tian Chang

**Affiliations:** ^1^ Academy of Art and Design, Guangdong AIB Polytechnic, Guangzhou, China; ^2^ School of Psychology, South China Normal University, Guangzhou, China; ^3^ University International College, Macau Unversity of Science and Technology, Macau, Macau SAR, China; ^4^ The National Research Institute for Teaching Materlals for Hong Kong, Macau and Taiwan, South China Normal University, Guangzhou, China; ^5^ Faculty of Education, Henan Normal University, Xinxiang, China; ^6^ Institute of International and Comparative Education, South China Normal University, Guangzhou, China

**Keywords:** smartphone addiction, academic procrastination, self-control, academic self-efficacy, college students

## Abstract

**Introduction:**

Researches have highlighted the individual roles of smartphone addiction (SA), self-control (S-C), and academic self-efficacy (AS-E) in predicting academic procrastination (AP), but studies on how these variables combine to affect AP are scarce. Drawing inspiration from the conceptual model of procrastination, this research endeavors to examine a serial mediation model in which SA serves as a precursor, with S-C and AS-E acting as serial mediators in predicting AP among college students who are prone to it.

**Methods:**

Participants were 1269 Chinese undergraduates (989 females, Mage = 19.03±1.00) from seven major regions in China. Data were collected using an online questionnaire. Descriptive and mediation analyses were conducted in SPSS 25.0.

**Results:**

The findings revealed that SA is indirectly linked to AP through independent and sequential mediation by S-C and AS-E. People with high SA reported lower levels of S-C, which in turn was associated with lower AS-E, and these were associated with higher reports of AP.

**Discussion:**

These findings offer significant contributions to the current body of research on AP, laying the groundwork for the development of interventions focused on reducing AP among college students.

## 1 Introduction

Academic procrastination (AP) is a pervasive phenomenon characterized by an individual’s deliberate postponement of academic tasks despite full awareness of their responsibility to complete them promptly (1). This behavior poses a significant concern among college students, as it is marked by an alarmingly high incidence rate and its detrimental, long-lasting impacts on personal and academic development. Among Chinese college students, a significant majority (74%) exhibit patterns of AP (2). Recent studies have shown that AP hinders academic success by increasing academic stress (3) and eroding dedication to learning (4), and it also correlates with reduced school connectedness, life satisfaction (5), and subjective well-being (6). Consequently, addressing AP is essential for cultivating better academic outcomes and promoting a healthier, more fulfilling student experience.

### 1.1 Theoretical background

The conceptual model of procrastination (7) integrates prominent psychological factors influencing procrastination into a structured framework. It categorizes these factors into three groups: task-related, personality-related, and other dynamic variables. Task-related factors include frustration, self-efficacy, and similar factors that shape how individuals perceive and approach tasks. For instance, lower self-efficacy tends to increase procrastination by undermining confidence in task completion. Personality-related elements, such as impulsiveness, affect decision-making processes. High impulsiveness is linked to greater procrastination due to a preference for immediate rewards over long-term goals. Dynamic variables include ego depletion, temptations, and other factors that divert attention from goal-directed activities. This model provides a theoretical framework for understanding the complex mechanisms of procrastination and lays the foundation for subsequent empirical research.

While previous studies on AP have primarily focused on task-related and personality-related factors (8), less attention has been given to other factors. For example, extensive research has demonstrated the role of academic stress (task-related factors), perfectionism (personality-related factors), and self-esteem (personality-related factors) in contributing to AP (8–10). Despite the limited focus on other factors in earlier research, the temptations posed by smartphones and related variables has been increasingly recognized as playing a critical role in AP (11, 12). Due to the diverse functionalities, real-time interactivity, and ease of operation of smartphones, they have become a significant temptation in the daily study and lives of college students (13). If college students are unable to resist the temptation of smartphones, excessive use can easily lead to SA. SA refers to a potent and enduring craving for and reliance on smartphones, leading to excessive engagement in phone-mediated activities that markedly impair individuals’ social and psychological well-being (14). Studies have indicated that SA can disrupt the learning process, consume valuable study time and effort, and inevitably hinder academic engagement and lead to AP (15, 16). Using the decision tree algorithm, Song and colleagues (17) found that SA is a significant factor in predicting AP among Chinese college students.

Under the framework of the conceptual model of procrastination (7), researchers have separately explored the impacts of other factors (e.g., SA) (17), personality-related factors (e.g., self-control, S-C) (18), and task-related factors (e.g., academic self-efficacy, AS-E) (19) on AP. However, how these factors interact with each other to influence AP remains unclear. Based on this, the present study aims to further explore the specific roles of S-C and AS-E in connecting SA with AP.

### 1.2 The mediating effect of self-control

S-C, which refers to the ability to consciously regulate automatic responses and align behavior with personal standards and long-term goals, is a positive trait that evolves throughout life (20). Its benefits have been well documented in terms of facilitating response inhibition, resisting temptation, and achieving academic success (21, 22). According to the reinforcement pathway of the I-PACE model, excessive Internet/smartphone use weakens inhibitory control (e.g., S-C), resulting in further negative consequences (23). This model suggest that S-C may mediate the relationship between SA and AP.

SA can contribute to a decline in S-C. Researchers have revealed that the addictive nature of smartphone use disrupts the neural mechanisms essential for maintaining S-C, particularly in areas of the brain responsible for inhibitory control and cognitive regulation (24). SA can divert attention and reduce cognitive control, causing the cognitive control system to become “idle” and leading to a decrease in S-C (25, 26). Recent studies have also shown that individuals with more severe SA exhibit poorer S-C (26, 27). Poor S-C is also a primary triggering factor for AP (28). Individuals with inadequate S-C often prioritize immediate rewards—such as the instant gratification provided by smartphones—over future benefits, such as completing academic assignments or studying for exams. This tendency to favor short-term pleasures over long-term goals is a hallmark of procrastinatory behavior (28). This deficit in S-C hinders students’ ability to focus on their current learning tasks, making them susceptible to distractions from surrounding stimuli and ultimately leading to AP (29). Empirical studies have revealed a negative connection between S-C and AP (18, 21). Taking into account both theoretical insights and empirical findings, this study proposes Hypothesis 1: S-C can mediate the relationship between SA and AP.

### 1.3 The mediating effect of academic self-efficacy

AS-E represents students’ confidence in their ability to achieve particular educational objectives effectively (30). It is an important proximal factor that influences academic motivation, engagement, and procrastination (19, 31). Self-efficacy not only promotes positive expectations for task outcomes but also reduces negative experiences during the task process, thereby decreasing procrastination (32, 33). Furthermore, self-efficacy plays a crucial role in shaping an individual’s approach to task initiation and persistence (34). Students who possess robust AS-E are more inclined to embark on and persevere through learning tasks, driven by their confidence in their abilities. Conversely, those with diminished AS-E, plagued by doubts about their capacity to complete such tasks, often struggle to initiate or maintain their efforts, leading to AP (35). Multiple studies have consistently demonstrated the negative relationship between AS-E and AP (19, 33, 36).

Research has shown that smartphone use serves as a good indicator for predicting self-efficacy among college students (37). Excessive smartphone usage, particularly when it becomes problematic, has been shown to negatively impact students’ learning capabilities, memory retention, and overall academic adaptability, thereby undermining their confidence in their academic competence (38, 39). For instance, a study involving 821 university students revealed a negative correlation between problematic smartphone behavior and students’ perceptions of their academic effectiveness, suggesting that overreliance on smartphones can erode their belief in their ability to succeed academically (37). According to Tuckman and Sexton, self-beliefs serve as a bridge between external factors and self-regulatory performance (40). Consistent with this perspective, recent research has found that AS-E acts as a mediator between smartphone use and AP (19, 36). Consequently, this study puts forward Hypothesis 2: AS-E mediates the relationship between SA and AP.

### 1.4 The serial mediating effect of self-control and academic self-efficacy

S-C is closely associated with AS-E. Numerous studies have examined how self-efficacy and S-C sequentially mediate between external influences and problematic behaviors (41, 42). However, no research has yet explored the serial mediation role of S-C and AS-E in this context. Based on the cyclical model of self-regulated learning (43), self-directed learning operates in a continuous loop, with S-C also influencing the formation of AS-E. Empirical evidence suggests that individuals who have stronger S-C generally exhibit greater self-efficacy (44, 45). They typically set and pursue long-term and valuable goals, living a more planned life, which contributes to enhancing their AS-E (46). Moreover, their ability to successfully resist temptations and avoid short-term detrimental behaviors aids them in achieving their goals and accumulating successful experiences, thereby further boosting their self-efficacy (47). Considering the theoretical and empirical evidence regarding the relationship between S-C and AS-E, this study proposes Hypothesis 3: S-C and AS-E have a serial mediation effect on the link between SA and AP.

### 1.5 The current study

There remains a lack of clarity regarding how SA, S-C, and AS-E collectively influence AP among college students. Drawing inspiration from the conceptual model of procrastination (7), and based on the reinforcement pathway of the I-PACE model (23) and the cyclical model of self-regulated learning (43), this study aims to establish a serial mediation model to test the three hypotheses mentioned above.

## 2 Materials and methods

### 2.1 Participants and procedure

Following approval from the Ethics Committee of School of Education, South China Normal University, this study was conducted via an online questionnaire on the Wenjuanxing platform. Prior to participation, the researchers read the informed consent form out loud to the participants, outlining the confidentiality of all study-related information and its exclusive use for research purposes. Participants can choose for themselves whether to participate or not to participate in the online survey. Upon acknowledging and consenting to these terms, participants proceeded to complete the survey. This cross-sectional study. A total of 1,505 questionnaires were collected. Following the exclusion of inattentive responses (including patterned answering and completion times under 100 seconds), 1,269 valid responses were retained. The final group of participants consisted of 1,269 university students (989 females and 280 males). Participants’ ages ranged from 17 to 24 years (*M* = 19.03, *SD* = 1.00). Additional demographic details are presented in **Table 1**.

**Table 1:** Demographic characteristics of participants.

Variables	Groups	N (%)
Major	Humanities and Social Sciences	669 (52.72%)
	Science/Medical Sciences and Engineering	354 (27.90%)
	Agriculture and Forestry	99 (7.80%)
	Arts	147 (11.58%)
Only child	Yes	164 (12.92%)
	No	1105 (87.08%)
Father’s education level	High school or below	1074 (84.63%)
	College or above	195 (15.37%)
Mother’s education level	High school or below	1101 (86.76%)
	College or above	168 (13.24%)

### 2.2 Measures

#### 2.2.1 Smartphone addiction

Smartphone Addiction Scale–Short Version (SAS-SV) is a scale for smartphone addiction that consisted of 10 items (48). It includes five sub-factors: daily-life disturbance, withdrawal, cyberspace-oriented relationship, overuse, and tolerance. Students in our study completed the Chinese version of SAS-SV (49). They rated their agreement with 10 statements on a scale of 1 to 6. All item employ positive scoring. As the mean score increases, the levels of SA intensify. In this study, Cronbach’s *α* of this scale was 0.92.

#### 2.2.2 Self-control

Participants completed a self-control scale that is widely applicable to Chinese college students (50). This scale was adapted by Chinese scholars from the Brief Self-Control Scale (51). It includes two sub-factors: impulse control (4 items), and self-discipline (3 items). Students report how well each item describes themselves on a scale of 1 to 5. Items 2, 4, 6 and 7 employ reverse scoring. As the mean score increases, the levels of S-C intensify. In this study, Cronbach’s *α* of this scale was 0.75.

#### 2.2.3 Academic self-efficacy

A 9-item Academic Self-Efficacy Scale (AS-ES), widely applicable to Chinese college students (52) was used to measure participants’ AS-E levels. This scale was translated by Chinese scholars from Pintrich’s and De Groot’s original AS-ES (53). Participants responded by indicating their level of agreement with the 9 statements on a scale of 1 to 5. This scale is a unidimensional measure with no reverse-scored items. As the mean score increases, the levels of AS-E intensify. In this study, Cronbach’s *α* of this scale was 0.92.

#### 2.2.4 Academic procrastination

College students completed the Academic Procrastination Inventory (API), which was translated by Chinese scholars (54), originating from Aitken (55). API is widely used in Chinese college students (54). Participants indicated whether each of the 19 procrastination behaviors applies to their own circumstances on a scale of 1 to 5. As a unidimensional measure, this scale contains 19 items, among which Items 2, 4, 7, 11, 12, 14, 16, 17, and 18 require reverse scoring. As the mean score increases, the levels of AP intensify. In this study, Cronbach’s *α* of this scale was 0.85.

#### 2.2.5 Control variables

Based on the findings of previous studies (3, 56), gender, age, and parental education levels may affect AP. This study added these three factors as control variables in the statistical analysis. Gender is dummy coded as 1 for males and 0 for females. Father’s and mother’s educational levels were evaluated across eight categories, from the lowest (uneducated) to the highest (PhD).

### 2.3 Data Analysis

**Figure 1:**
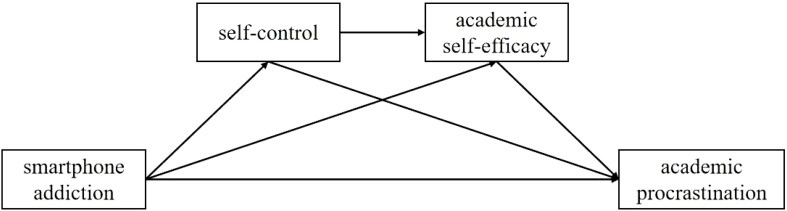
The hypothesized serial mediation model.

We first carried out a preliminary assessment for potential common method variance, followed by descriptive and correlational analyses with SPSS 25.0. Our hypothesized serial mediation model was tested via PROCESS Macro Model 6 (57). The hypothesized serial mediation model is presented in **Figure 1**. Utilizing bootstrapping (n = 5000), a technique particularly suitable for testing mediation models without assuming normality in the data, we further investigated the significance of the mediation effect.

## 3 Results

### 3.1 Basic analysis

Before conducting mediation analysis, we perform a basic analysis. First, the results of the Harman single-factor test indicate that the amount of variation explained by the first factor is 27.11%, which is less than 40% (58). Therefore, this study does not suffer from serious common method bias. Second, the results of correlation analyses revealed that SA has a negative link with S-C and AS-E, but a positive link with AP. S-C has a positive correlation with AS-E and a negative link with AP. Moreover, AS-E also has a negative link with AP (see **Table 2**).

**Table 2:** Descriptive statistics and correlations among main variables.

Variables	*M*	*SD*	1	2	3	4
1. Smartphone addiction	2.72	0.97	1.00			
2. Self-control	3.44	0.58	−0.37***	1.00		
3. Academic self-efficacy	3.32	0.58	−0.55***	0.42***	1.00	
4. Academic procrastination	2.50	0.48	0.46***	−0.42***	−0.61***	1.00

***p < 0.001.

### 3.2 Mediating analysis

After accounting for control variables, our results, presented in **Tables 3**, **4**, revealed a negative association between SA and S-C (*β* = −0.63, *p* < 0.001) as well as between SA and AS-E (*β* = −0.21, *p* < 0.001). Furthermore, S-C (*β* = −0.46, *p* < 0.001) and AS-E (*β* = −0.17, *p* < 0.001) demonstrated a negative association with AP. Bootstrapping results further confirmed the significance of S-C’s (effect size = 0.29, 95% CI [0.24, 0.34]) and AS-E’s (effect size = 0.04, 95% CI [0.02, 0.05]) mediation in the SA-AP relationship. Notably, S-C exhibited a positive correlation with AS-E (*β* = 0.31, *p* < 0.001). Moreover, the serial mediation effect of S-C and AS-E in the SA-AP connection was found to be significant, with an effect size of 0.03 (95% CI [0.02, 0.05]), as indicated by the bootstrapping results. Given that gender imbalance may potentially influence the results of this study, we conducted a gender-specific analysis to examine the robustness of the findings. The results indicated that the chain mediation effect of S-C and AS-E was significant for both male and female students (males: effect size = 0.03, 95% CI [0.01, 0.04]; females: effect size = 0.03, 95% CI [0.01, 0.04]), as presented in **Supplementary Table S1-S3**. These findings suggest that the observed chain mediation effects are stable across genders within the study sample.

**Table 3:** Results of the serial mediation model.

Independent variables	Dependent variables					
	Self-control		Academic self-efficacy		Academic procrastination	
	*β*	*t*	*β*	*t*	*β*	*t*
Gender	0.01	0.08	0.16	2.67**	0.10	1.94
Age	−0.01	−0.53	0.01	0.36	0.06	2.37**
Father’s education level	−0.08	−2.44*	0.07	2.16*	−0.06	−2.05*
Mother’s education level	0.06	1.84	0.04	1.35	0.03	0.91
Smartphone addiction	−0.63	−22.97***	−0.21	−6.19***	0.17	5.66***
Self-control			0.31	10.36***	−0.46	−17.45***
Academic self-efficacy					−0.17	−6.85***
*R^2^*	0.30		0.22		0.43	
*F*	108.84***		57.63***		132.92***	

*p < 0.05, **p < 0.01, ***p < 0.001.

**Table 4:** The mediating role of self-control and academic self-efficacy.

	Effect	Boot SE	Boot LLCI	Boot ULCI
Direct effect	0.17	0.03	0.11	0.23
Total indirect effect	0.36	0.03	0.31	0.41
Indirect effect 1	0.29	0.03	0.24	0.34
Indirect effect 2	0.04	0.01	0.02	0.05
Indirect effect 3	0.03	0.01	0.02	0.05

Indirect effect 1, smartphone addiction→self-control→academic procrastination; Indirect effect 2, smartphone addiction→academic self-efficacy→academic procrastination; Indirect effect 3, smartphone addiction→self-control→academic self-efficacy→academic procrastination; LLCI, lower limit of the confidence interval; ULCI, upper limit of the confidence interval.

## 4 Discussion

A serial mediation framework was constructed to assess the independent mediation roles of S-C and AS-E, along with their sequential mediation effect, in linking SA to AP. In this study, all hypotheses have been well validated. The results revealed that SA was indirectly associated with AP through both independent and sequential mediation by S-C and AS-E. Specifically, higher SA correlated with lower S-C and AS-E, which in turn were linked to higher AP. These findings align with the conceptual model of procrastination (7) and extend prior research by integrating SA, S-C, and AS-E into a unified framework.

Consistent with Hypothesis 1, our study uncovered a significant mediation effect of S-C in the connection between SA and AP. This result supports the reinforcement pathway of the I-PACE model (23), suggesting that S-C may play an important role in the relationship between SA and AP. Previous studies have also identified S-C as a crucial internal psychological mechanism through which problematic smartphone use or Internet addiction influences students’ procrastination behaviors (18, 59). For example, a longitudinal study of 622 college students found that S-C could significantly mediate the link between problematic smartphone use and sleep procrastination (59). The strength model of S-C (20) indicated that S-C requires the expenditure of individuals’ limited psychological resources, such that depletion of these resources in one domain leads to a corresponding decrease in available S-C strength in other domains. Individuals addicted to smartphones may need these resources to suppress impulses when faced with smartphone temptations (21). Reduced S-C capacity could potentially lead to difficulties in sustaining focus on learning tasks, potentially contributing to AP (18, 29).

Supporting Hypothesis 2, our research revealed that AS-E plays a significant intermediary role in the connection between SA and AP. This result tallies with earlier research findings (19, 36), which have identified AS-E as a key factor linking smartphone usage to academic procrastination. Previous research has highlighted the importance of AS-E in predicting academic performance and behavioral outcomes (35). In the context of SA, students who exhibit higher levels of SA tend to report lower levels of AS-E (15, 37). This association may reflect that excessive smartphone use can interfere with study habits and routines, potentially reducing students’ confidence in their ability to succeed academically. Other studies also found that individuals with high AS-E often exhibit a stronger belief in their capacity to manage academic tasks effectively, making them more inclined to take the initiative and complete them on time, thereby reducing their propensity for procrastination (36). Conversely, college students who lack of confidence may doubt their capacity to complete academic tasks, prompting them to procrastinate before starting these tasks (35).

Another crucial finding of this study is that S-C and AS-E serially mediated the relationship between SA and AP, aligning with Hypothesis 3. This discovery resonates with the cyclical model of self-regulated learning (43), indicating that S-C is positively correlated with AS-E. Moreover, our findings are consistent with previous research, which highlight that individuals with strong S-C are better equipped to regulate their behaviors, resist distractions, and maintain focus on long-term academic goals, which may enhance their confidence in their ability to succeed academically (44, 45). However, SA may undermines S-C by depleting students’ cognitive resources and making them more susceptible to external distractions (26, 47). This study suggests that higher levels of S-C are associated with greater confidence in one’s ability to succeed academically (AS-E), and may further escalate the propensity for AP (60). However, it should be noted that these interpretations are speculative and solely based on correlational data. While our findings align with existing theoretical frameworks and empirical evidence, the cross-sectional nature of this study limits our ability to establish causal relationships or determine the temporal sequence of these processes. Future longitudinal or experimental studies are necessary to validate these associations and clarify the directionality of the relationships between SA, S-C, AS-E, and AP.

There is evidence to confirm the relationship between SA, S-C, AS-E, and AP, respectively (17–19). However, how these factors interact with each other to influence AP is unclear. Based on the conceptual model of procrastination (7), this study integrated SA, S-C, AS-E, and AP into a serial mediation model to explore the underlying mechanisms through which SA influences AP among college students. The results indicate that S-C and AS-E serve as serial mediators between SA and AP. As well as enriching the content of the conceptual model of procrastination, this study also enhances understanding of the pathways through which SA, S-C, and AS-E jointly impact AP. The results imply that intervening in any single variable within the serial mediation — comprising S-C and AS-E — can disrupt the interconnected sequence. First, enhancing S-C is vital. Mindfulness training, particularly brief mindfulness meditation, has proven effective in rapidly developing S-C abilities, even in environments with limited resources (61, 62). By practicing mindfulness meditation, students can strengthen their S-C mechanisms, making them less prone to smartphone distractions and more proficient in managing their academic responsibilities efficiently. In addition to enhancing S-C, interventions targeting AS-E may also play a significant role in breaking the serial mediation chain. Self-care programs based on the Orem model have been shown to effectively improve AS-E (63).

Limitations that merit consideration are as follows. First, the data collected solely relied on participants’ self-reports, potentially introducing social desirability bias. To enhance the accuracy of assessments of students’ SA and AP, future research could integrate reports from teachers and parents. Second, cross-sectional design was unable to establish causality between the examined variables. To rigorously explore the chained mediation effects of S-C and AS-E in the association between SA and AP, future research could adopt longitudinal or cross-lagged approaches. Third, while the procrastination conceptual model (7) categorizes influential factors into task-related, personality-related, and other factors, this study confined its focus to the mechanisms through which SA, S-C, and AS-E impact AP. Future studies could broaden the scope to investigate the mechanisms through which task-related factors such as autonomous motivation, personality-related factors such as impulsiveness, and other factors such as mood influence AP. Finally, although this study conducted gender-specific analyses and found that gender imbalance did not affect the stability of the results, future research would benefit from using samples with a more balanced proportion of male and female participants.

## Data Availability

The raw data supporting the conclusions of this article will be made available by the authors, without undue reservation.
