# Supplementary material for: Smartphone addiction and academic procrastination among college students: a serial mediation model of self-control and academic self-efficacy
Source: Front Psychiatry. 2025 May 29;16:1572963. doi: 10.3389/fpsyt.2025.1572963 (PMC12158977; doi:10.3389/fpsyt.2025.1572963)
Supplement: Supplementary file 1 [file Table1.docx]

**Table S1. Results of the serial mediation model in males**

| **Independent variables** | **Dependent variables** | | | | | |
| --- | --- | --- | --- | --- | --- | --- |
|  | **Self-control** | | **Academic self-efficacy** | | **Academic procrastination** | |
|  | ***β*** | ***t*** | ***β*** | ***t*** | ***β*** | ***t*** |
| Age | 0.01 | 0.51 | 0.03 | 0.97 | 0.05 | 1.95 |
| Father’s education level | −0.06 | −1.74 | 0.07 | 2.10* | −0.06 | −1.84 |
| Mother’s education level | 0.08 | 2.27* | 0.02 | 0.68 | 0.02 | 0.57 |
| Smartphone addiction | −0.58 | −19.35*** | −0.19 | −5.13*** | 0.17 | 5.12*** |
| Self-control |  |  | 0.31 | 9.14*** | −0.49 | −15.80*** |
| Academic self-efficacy |  |  |  |  | −0.15 | −5.20*** |
| *R^2^* | 0.28 | | 0.20 | | 0.42 | |
| *F* | 97.35*** | | 47.99*** | | 117.08*** | |

**p* < 0.05, ****p* < 0.001.

**Table S2. Results of the serial mediation model in females**

| **Independent variables** | **Dependent variables** | | | | | |
| --- | --- | --- | --- | --- | --- | --- |
|  | **Self-control** | | **Academic self-efficacy** | | **Academic procrastination** | |
|  | ***β*** | ***t*** | ***β*** | ***t*** | ***β*** | ***t*** |
| Age | −0.01 | −0.51 | 0.03 | 0.97 | 0.05 | 1.95 |
| Father’s education level | −0.06 | −1.74 | 0.07 | 2.10* | −0.06 | −1.84 |
| Mother’s education level | 0.08 | 2.27* | 0.02 | 0.68 | 0.02 | 0.57 |
| Smartphone addiction | −0.58 | −19.35*** | −0.19 | −5.13*** | 0.17 | 5.12*** |
| Self-control |  |  | 0.31 | 9.14*** | −0.49 | −15.80*** |
| Academic self-efficacy |  |  |  |  | −0.15 | −5.20*** |
| *R^2^* | 0.28 | | 0.20 | | 0.42 | |
| *F* | 97.35*** | | 47.99*** | | 117.08*** | |

**p* < 0.05, ****p* < 0.001.

**Table S3. The mediating role of self-control and academic self-efficacy in males and females**

|  | **Effect** | **Boot SE** | **Boot LLCI** | **Boot ULCI** |
| --- | --- | --- | --- | --- |
| **males** | | | | |
| Direct effect | 0.17 | 0.03 | 0.11 | 0.24 |
| Total indirect effect | 0.34 | 0.03 | 0.28 | 0.40 |
| Indirect effect 1 | 0.29 | 0.03 | 0.23 | 0.34 |
| Indirect effect 2 | 0.03 | 0.01 | 0.01 | 0.05 |
| Indirect effect 3 | 0.03 | 0.01 | 0.01 | 0.04 |
| **females** | | | | |
| Direct effect | 0.17 | 0.03 | 0.11 | 0.24 |
| Total indirect effect | 0.34 | 0.03 | 0.28 | 0.40 |
| Indirect effect 1 | 0.29 | 0.03 | 0.23 | 0.34 |
| Indirect effect 2 | 0.03 | 0.01 | 0.01 | 0.05 |
| Indirect effect 3 | 0.03 | 0.01 | 0.01 | 0.04 |

Indirect effect 1: smartphone addiction→self-control→academic procrastination. Indirect effect 2: smartphone addiction→academic self-efficacy→academic procrastination. Indirect effect 3: smartphone addiction→self-control→academic self-efficacy→academic procrastination. LLCI: lower limit of the confidence interval. ULCI: upper limit of the confidence interval.
